# Reactive oxygen species promotion drives auranofin’s antiviral activity against hepatitis E virus

**DOI:** 10.1128/jvi.01917-25

**Published:** 2025-12-09

**Authors:** Kateland Tiller, S. Tyler Williams, Bo Wang, Debin Tian, Xiang-Jin Meng, James Weger-Lucarelli

**Affiliations:** 1 Virginia-Maryland College of Veterinary Medicine, Department of Biomedical Sciences and Pathobiology, Virginia Polytechnic Institute and State University 229659, Blacksburg, Virginia, USA; Wake Forest University School of Medicine, Winston-Salem, North Carolina, USA

**Keywords:** drug repurposing, auranofin, reactive oxygen species, hepatitis E virus, antiviral

## Abstract

Hepatitis E virus (HEV) causes roughly 20 million yearly global infections and is associated with chronic hepatitis, neurological sequelae, and pregnancy-related adverse outcomes that require antiviral intervention. While there are no approved HEV-specific therapeutics, ribavirin and pegylated interferon, prescribed off-label, remain the current standard of care. However, ribavirin resistance and toxicity highlight the unmet clinical need to identify safer, HEV-specific antivirals. Here, we identify reactive oxygen species (ROS) promotion as a previously unrecognized host-directed antiviral mechanism against HEV, revealed through the activity of the FDA-approved drug auranofin. Auranofin, which is known to elevate intracellular ROS, displays antiviral activity against several viruses. We revealed here that auranofin exhibits robust, dose-dependent antiviral activity against two clinically relevant HEV genotypes and a ribavirin treatment failure-associated mutant. ROS inhibition reversed auranofin-mediated ROS promotion and antiviral activity, establishing a mechanistic link between ROS promotion and antiviral activity. Treatment with D-amino acid oxidase, which breaks down D-amino acids producing the ROS H_2_O_2_, exerted dose-dependent anti-HEV activity. This effect was reversed by ROS inhibition, demonstrating that ROS accumulation alone is sufficient for antiviral activity. We also revealed that ROS promotion by auranofin drives activation of antioxidant, ER stress, and interferon-stimulated gene expressions, further supporting induction of ROS-dependent antiviral signaling. Lastly, we demonstrated that combined treatment with auranofin and ribavirin exhibits synergistic antiviral activity *in vitro*. These findings highlight the promotion of ROS as a previously underappreciated host-directed antiviral mechanism and support the repurposing of auranofin—alone or in combination with ribavirin—as a therapeutic strategy against HEV.

**IMPORTANCE:**

Hepatitis E virus (HEV) lacks approved virus-specific antiviral therapies, and off-label treatments with ribavirin and pegylated interferon are limited by toxicity and emerging resistance mutants. This study identifies reactive oxygen species (ROS) promotion mediated by the FDA-approved drug auranofin and D-amino acid oxidase as an effective antiviral strategy against multiple genotypes of HEV, including two globally relevant human-associated genotypes and a ribavirin treatment failure-associated HEV mutant. The observed synergistic anti-HEV activity *in vitro* for combined treatment with both auranofin and ribavirin suggests a potential clinically effective combinational therapeutic approach. ROS promotion through auranofin or other means represents an underexplored antiviral strategy with potential for broad-spectrum activity against a range of viral diseases.

## INTRODUCTION

Hepatitis E virus (HEV) is a globally distributed virus that has been estimated by the World Health Organization (WHO) to cause approximately 20 million infections every year, with 3.3 million symptomatic infections and approximately 44,000 deaths, making HEV a major cause of acute and chronic viral hepatitis worldwide (1–3). Most HEV infections in developing countries are acquired through virus-contaminated water during large-scale outbreaks. In contrast, in industrialized countries with good sanitary infrastructure, cases often result from zoonotic transmission through consumption of undercooked or raw animal meat products (4, 5). Based on these multiple routes of transmission, it is suggested that the actual global disease burden caused by HEV is greatly underestimated (6, 7).

HEV is a single-stranded positive-sense RNA virus belonging to the family *Hepeviridae*, which consists of two subfamilies: *Orthohepevirinae* and *Parahepevirinae* (8). The major HEV genotypes known to infect humans belong to the species *balayani* in the genus *Paslahepevirus*. Genotypes 1 and 2 (HEV-1 and HEV-2) exclusively infect humans and are responsible for large-scale outbreaks in developing countries. In contrast, HEV-3 and HEV-4 infect both humans and other animals, causing sporadic cases of zoonotic transmission (9). HEV is a non-enveloped, spherical virus with particles of approximately 30–35 nm in stool samples, although virions circulating in the blood of infected individuals and those produced in cell culture exist as quasi-enveloped particles (10–13). The HEV genome is approximately 7.2 kb in length, containing three partially overlapping open reading frames (ORFs): ORF1 encodes non-structural proteins responsible for replication, ORF2 encodes the structural capsid protein, and ORF3 encodes a small protein involved in virus replication and assembly (14–17).

HEV poses a significant global health burden due to its varied clinical manifestations and potential for severe disease in vulnerable populations. While most patients experience a self-limiting, acute infection, HEV infection can also result in chronic and/or deadly outcomes in at-risk groups, including immunocompromised people, pregnant women, or those who have pre-existing liver disease (18–20). Immunocompromised individuals, such as those who are HIV-positive, solid organ transplant recipients, and patients undergoing chemotherapy treatments, are more likely to develop chronic HEV infections, which can become deadly if liver fibrosis or cirrhosis develops (21–23). Severe disease outcomes associated with HEV infection disproportionately affect pregnant women and their developing fetuses. HEV infection in the second or third trimesters of pregnancy greatly increases the risk of developing fulminant hepatic failure (FHF) and death (19, 24). Vertical transmission has also been reported, resulting in adverse fetal outcomes of preterm delivery, fetal distress, and/or low birth weight (25), although others failed to transmit HEV vertically under experimental conditions (26, 27). A significant proportion of HEV-infected individuals develop various neurological sequelae, including Guillain-Barré syndrome and neuralgic amyotrophy (28–30). Recently, HEV has been recognized as the third leading cause of foodborne viral illness (31), and pork is a leading source of foodborne HEV infections (32–34). Chronic hepatitis E, HEV-associated neurological complications, foodborne hepatitis E, and the severity of disease outcomes in certain at-risk populations highlight the urgent need for effective HEV-specific antivirals.

Currently, no therapeutics are approved for treating hepatitis E, and the only hepatitis E vaccines approved for use are in China (35) and Pakistan (36). Ribavirin and pegylated interferon are used as off-label treatments and represent the current standard of care for treating HEV infection. However, ribavirin resistance has been reported (37, 38), and pegylated interferon is associated with significant side effects (39–41), highlighting the need for safer and more effective virus-specific antivirals to treat hepatitis E. Reflecting this importance, in 2024, the WHO listed HEV-3 as a prototype pathogen for the *Hepeviridae* family (42). Prototype pathogens are designated to accelerate pandemic preparedness by serving as representative viruses from high-risk families, through which broadly applicable antivirals, vaccines, and diagnostic tools can be developed and later adapted to novel emerging viral threats. Therefore, antiviral testing against HEV is significant not only for identifying urgently needed treatment options for HEV infections, but also for contributing to global preparedness efforts against future viral outbreaks.

Recently, there has been an increasing interest in repurposing FDA-approved drugs as antivirals. This process is faster, safer, and more cost-effective than identifying and developing novel antivirals (43, 44). One such FDA-approved drug is auranofin, a gold-based compound used in patients with rheumatoid arthritis. Auranofin has shown promise in treating a wide range of ailments, including viral, bacterial, and fungal infections as well as cancer (45). Notably, it has demonstrated antiviral activity against chikungunya virus (46), human immunodeficiency virus (47, 48), and SARS-CoV-2 (49, 50). However, auranofin’s antiviral mechanism remains poorly defined, and its efficacy against HEV has not been examined. We tested the antiviral activity of auranofin against clinically relevant genotypes of HEV using a human hepatocyte cell line. We found that auranofin displayed dose-dependent antiviral activity against HEV at non-toxic concentrations. Further studies into the antiviral mechanism of action revealed that reactive oxygen species (ROS) mediate auranofin’s antiviral activity and that promoting ROS alone displayed robust antiviral activity against HEV. Transcriptional analyses also revealed ROS-promoted modulation of antioxidant, ER stress, and interferon-stimulated pathways, supporting ROS-driven antiviral activity. Furthermore, we also demonstrated that combined treatment with ribavirin and auranofin yields a synergistic antiviral effect, underscoring the potential for more effective combinational therapies for chronic hepatitis E and HEV-associated neurological sequelae. Collectively, our results suggest that ROS promotion is a promising host-directed antiviral strategy against HEV infection.

## RESULTS

### Auranofin displays dose-dependent antiviral activity against HEV-1 Sar55 Gluc replicon and HEV-1 infectious reporter virus Sar55(Hib)

**Fig 1:**
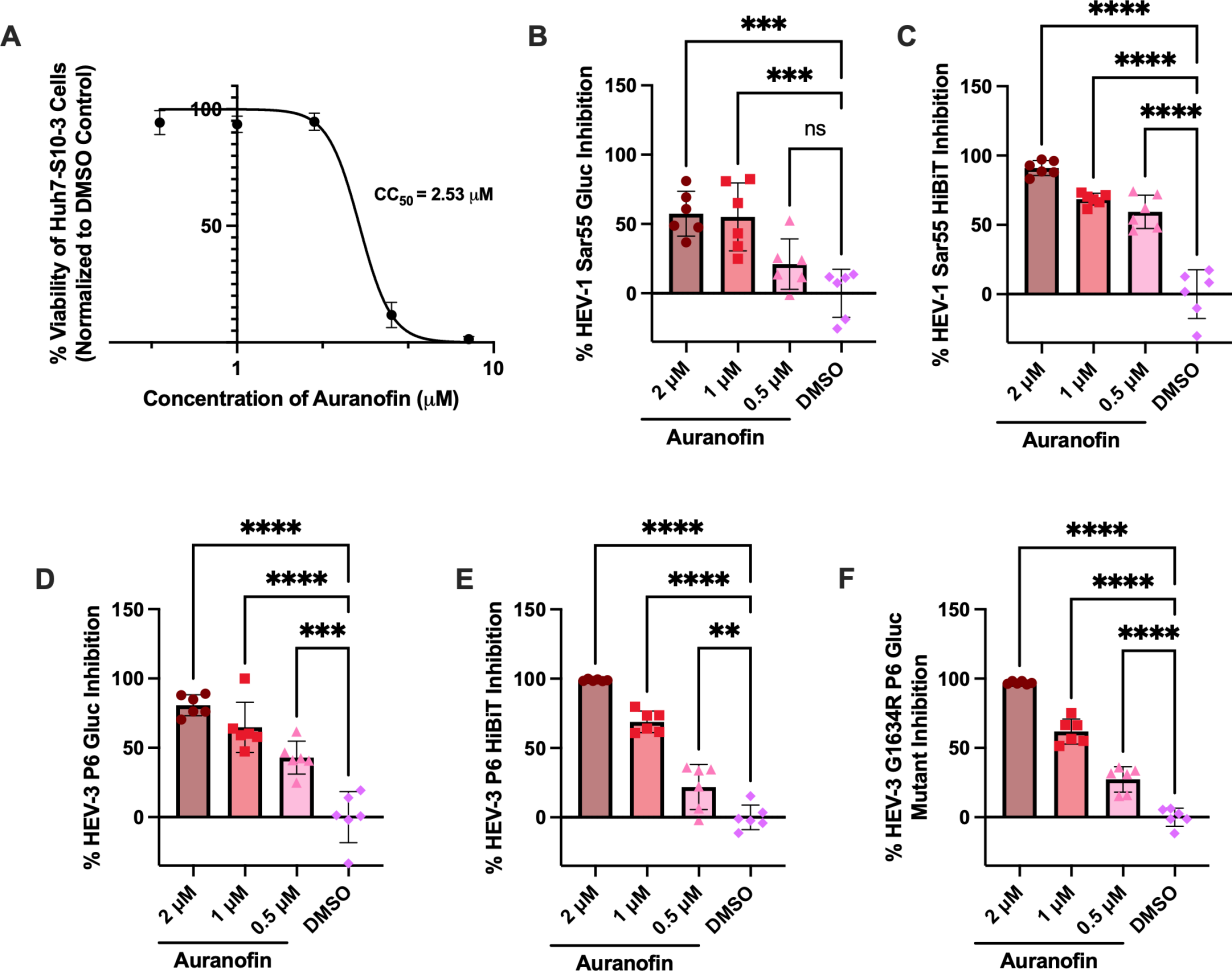
Auranofin displays dose-dependent antiviral activity at non-toxic concentrations against two genotypes of HEV and a ribavirin treatment failure-associated HEV mutant. (**A**) The effect of auranofin on cell viability in Huh7-S10-3 (human hepatocyte) cells, measured via MTS assay. Data was collected 72 h after the addition of the compound, background-subtracted, and normalized to the DMSO vehicle control. X-axis values are log-transformed. The lines represent non-linear regression curves, and data points represent means ± SD; *n* = 6 performed in two independent experiments. (**B and C**) Antiviral activity of auranofin against the HEV-1 Sar55 Gluc replicon (**B**) and HEV-1 Sar55 (Hib) infectious reporter virus (**C**). (**D–F**) Antiviral activity of auranofin against the HEV-3 P6 Gluc replicon (**D**), HEV-3 P6 (Hib) infectious reporter virus (**E**), and HEV-3 G1634R P6 Gluc ribavirin resistance mutant (**F**). For antiviral assays, data were collected 72 h post-transfection for Gluc replicons and the HEV-3 P6 (Hib) infectious reporter virus, and 7 days post-transfection for the Sar55 (Hib) infectious reporter virus. Data from panels **B** to **F** were background-subtracted, normalized to the DMSO level in the 2 μM auranofin group, and analyzed by one-way ANOVA with Dunnett’s multiple comparisons test (single pooled variance). Error bars represent ± SD; *n* = 6 performed in two separate experiments. ns = *P* > 0.05, ** = *P* < 0.01, *** = *P* < 0.001, **** = *P* < 0.0001.

To establish a range of non-toxic concentrations to test auranofin’s antiviral activity, cell viability was assessed 72 h post-compound application on Huh7-S10-3 cells via an MTS assay. At concentrations below 2 μM, auranofin displayed low toxicity in Huh7-S10-3 cells. Cell viability decreased at concentrations above 2 μM, resulting in a CC_50_ of 2.53 μM (Fig. 1A). Based on these results, we performed subsequent experiments using auranofin at concentrations of 2 μM or below.

To assess auranofin’s antiviral activity against HEV, we first tested non-toxic doses against the HEV-1 Sar55 Gluc replicon and the HEV-1 Sar55(Hib) HiBiT-tagged infectious reporter virus that enables luciferase-based detection of extracellular viral particles (51). The Gluc expression (72 h post-inoculation) and HiBiT expression (7 days post-inoculation) were measured to indicate HEV-1 replication. Auranofin exerts robust dose-dependent antiviral activity against the HEV-1 Sar55 Gluc replicon (Fig. 1B) and the HEV-1 Sar55(Hib) HiBiT-tagged infectious virus (Fig. 1C), demonstrating auranofin’s antiviral activity against a replicon and infectious HEV-1 system.

### Auranofin also displays antiviral activity against HEV-3 P6 Gluc replicon, HEV-3 infectious reporter virus P6(Hib), and a ribavirin treatment failure-associated HEV-3 P6 G1634R mutant

To determine if auranofin displays antiviral activity against other clinically relevant HEV genotypes, we assessed its activity against a genotype 3 HEV (HEV-3), a zoonotic genotype. As with HEV-1, auranofin displayed dose-dependent antiviral activity against the HEV-3 P6 Gluc replicon at non-toxic concentrations (72 h post-inoculation), resulting in an EC_50_ of 0.54 μM (Fig. 1D; Fig. S1). To assess auranofin’s efficacy against an HEV-3 P6 infectious virus, we constructed an HiBiT-expressing P6 infectious clone, HEV-3 P6(Hib), using a bacterial-free cloning approach (51–53). We showed that auranofin also displays dose-dependent antiviral activity against the HEV-3 P6(Hib) infectious virus (Fig. 1E). To determine if auranofin is effective against a ribavirin treatment failure-associated HEV mutant, antiviral testing was performed against an HEV-3 P6 Gluc mutant containing the G1634R mutation, which is associated with ribavirin treatment failure *in vivo* (37, 54). Similar to the WT HEV-3 P6 Gluc and HEV-3 P6 (Hib) infectious virus, the HEV-3 G1634R P6 Gluc mutant displayed susceptibility to auranofin treatment (Fig. 1F). Altogether, these results demonstrated that auranofin is an effective antiviral against replicon and infection systems of two different HEV genotypes, as well as a mutant associated with ribavirin treatment failure.

### ROS inhibitors, N-acetylcysteine (NAC) and dithiothreitol (DTT), reverse auranofin’s antiviral activity

Auranofin has been reported to inhibit glutathione peroxidase (GPx) and thioredoxin reductase (TrxR), resulting in increased intracellular ROS levels (55–58). ROS are chemically active byproducts of cellular metabolism that can play important roles in cell signaling pathways and immune activation (59). Thus, we hypothesized that ROS production may contribute to auranofin’s antiviral activity against HEV. To test this hypothesis, we treated cells with common ROS inhibitors, NAC and DTT, in the presence of auranofin. NAC reduces ROS by promoting glutathione production, directly scavenging ROS, and modulating redox-signaling pathways (60, 61). DTT is thought to neutralize ROS and other free radicals, in addition to protecting against mitochondrial oxidative damage and regenerating glutathione from oxidized glutathione (GSSG) (62, 63), suggestive of differential anti-ROS mechanisms for NAC and DTT.

**Fig 2:**
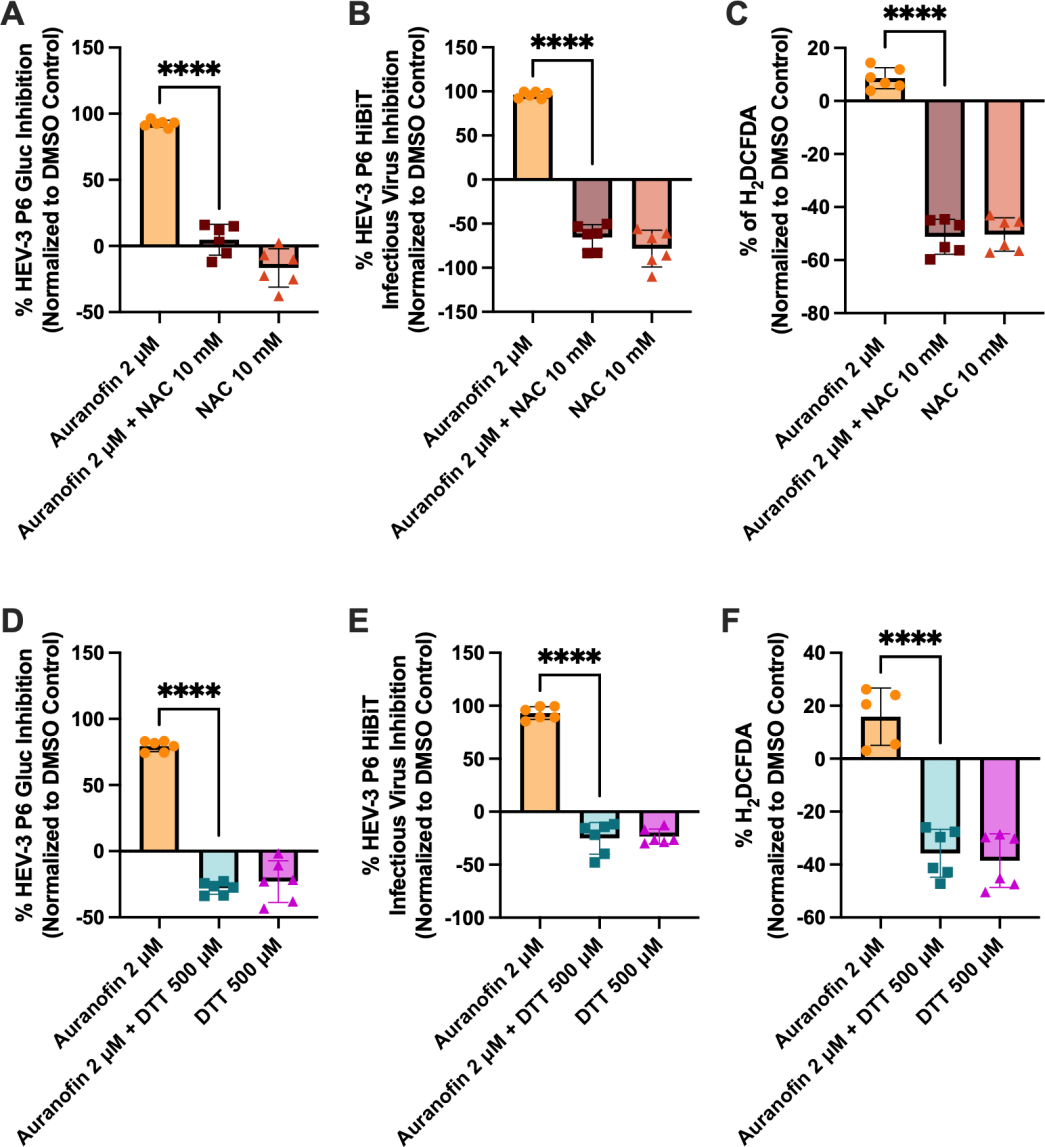
Auranofin’s antiviral activity is dependent on the promotion of reactive oxygen species. (**A–C**) The effect of 2 μM auranofin in the presence of 10 mM NAC against HEV-3 P6 Gluc replicon (**A**), HEV-3 P6 HiBiT infectious virus (**B**), and ROS production as quantified by the H_2_DCFDA fluorescent probe (**C**). (**D–F**) The effect of 2 μM auranofin in the presence of 500 μM DTT against HEV-3 P6 Gluc replicon (**D**), HEV-3 P6 HiBiT infectious virus (**E**), and ROS production as measured by the H_2_DCFDA fluorescent probe (**F**). Single-cell suspensions were analyzed by flow cytometry, and the median fluorescent peak was recorded. Data is background-subtracted, normalized to DMSO vehicle control, and analyzed by one-way ANOVA with Dunnett’s multiple comparisons test (single pooled variance). Data points represent means ± SD; *n* = 6 performed in two independent experiments. **** = *P* < 0.0001.

Co-treatment with 10 mM NAC and 2 μM auranofin completely reversed the antiviral activity of 2 μM auranofin against the HEV-3 P6 Gluc replicon (Fig. 2A) and the HEV-3 P6 HiBiT infectious virus (Fig. 2B). To validate the impact on intracellular ROS levels, we used the fluorescent probe H_2_DCFDA to measure general intracellular ROS levels. When compounds and the H_2_DCFDA probe were applied to Huh7-S10-3 cells, we observed an increase in ROS in the presence of 2 μM auranofin (Fig. 2C). This increase was reversed with the combined treatment of 10 mM NAC, mirroring the reversal of antiviral activity. NAC treatment resulted in a robust reversal of baseline ROS, which was normalized to the DMSO control, resulting in negative values. Importantly, DMSO increases ROS, likely accounting for the values below the baseline (64). To test the contribution of ROS in an orthogonal manner, we tested 500 μM DTT in the presence of 2 μM auranofin. Again, we observed a reversal of antiviral activity against the HEV-3 P6 Gluc replicon (Fig. 2D) and the HEV-3 P6 HiBiT infectious virus (Fig. 2E). ROS promotion was also reversed in the presence of DTT (Fig. 2F). Together, these results suggest that ROS promotion is necessary for auranofin’s antiviral effects against HEV.

### The ROS promoter D-amino acid oxidase (DAAO) displays antiviral activity against HEV

**Fig 3:**
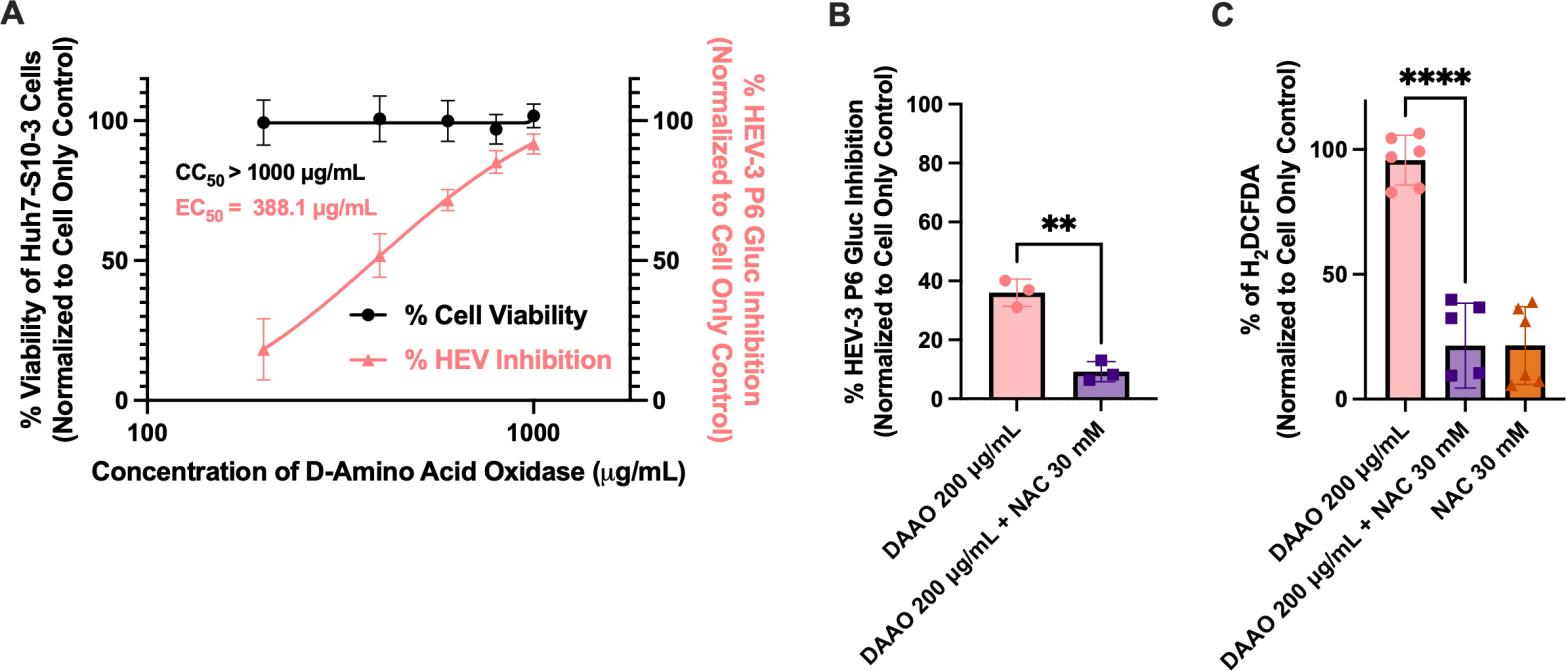
Independent ROS generation by DAAO suppresses HEV replication, demonstrating ROS sufficiency for antiviral activity. (**A**) The effect of DAAO on cell viability and antiviral activity of the HEV-3 P6 Gluc replicon in Huh7-S10-3 cells. Data collected 72 h post-compound application was background subtracted and normalized to the cell-only control. X-axis values are log-transformed. The lines represent non-linear regression curves, and data points represent means ± SD; *n* = 6 performed in two independent experiments. (**B**) The antiviral effect of 200 μg/mL DAAO in the presence of 30 mM NAC on the HEV-3 P6 Gluc replicon. (**C**) Generalized ROS measurement after 30-min incubation with 10 μM treatment of H_2_DCFDA fluorescence probe. Samples were also incubated for 30 min with 200 μg/mL DAAO and 30 mM NAC. Single-cell suspensions were analyzed via flow cytometry, and median fluorescent peak was recorded. Data from panels B and C are background-subtracted, normalized to cell-only control, and analyzed by one-way ANOVA with Dunnett’s multiple comparisons test (single pooled variance) with data points representing means ± SD. *n* = 6, performed in two independent experiments. ** = *P* < 0.01, **** = *P* < 0.0001.

To directly test whether ROS production alone is sufficient to induce antiviral activity, we tested the ROS promoter DAAO. DAAO specifically promotes hydrogen peroxide (H_2_O_2_) production through the breakdown of D-amino acids, a ROS-promoting mechanism distinct from auranofin and independent of GPx and TrxR inhibition (65). DAAO displayed strong antiviral activity against HEV-3 P6 Gluc and no cytotoxicity at the doses tested, with a CC_50_ of >1,000 µg/mL and an EC_50_ of 388.1 μg/mL (Fig. 3A). The antiviral activity (Fig. 3B) and ROS promotion (Fig. 3C) of 200 μg/mL DAAO were reversed by co-treatment with 30 mM NAC. These results identify that ROS promotion alone is sufficient for anti-HEV activity and identify a novel antiviral mechanism against HEV replication.

### Auranofin treatment induces ROS-dependent upregulation of antioxidant, ER stress, and antiviral transcripts

**Fig 4:**
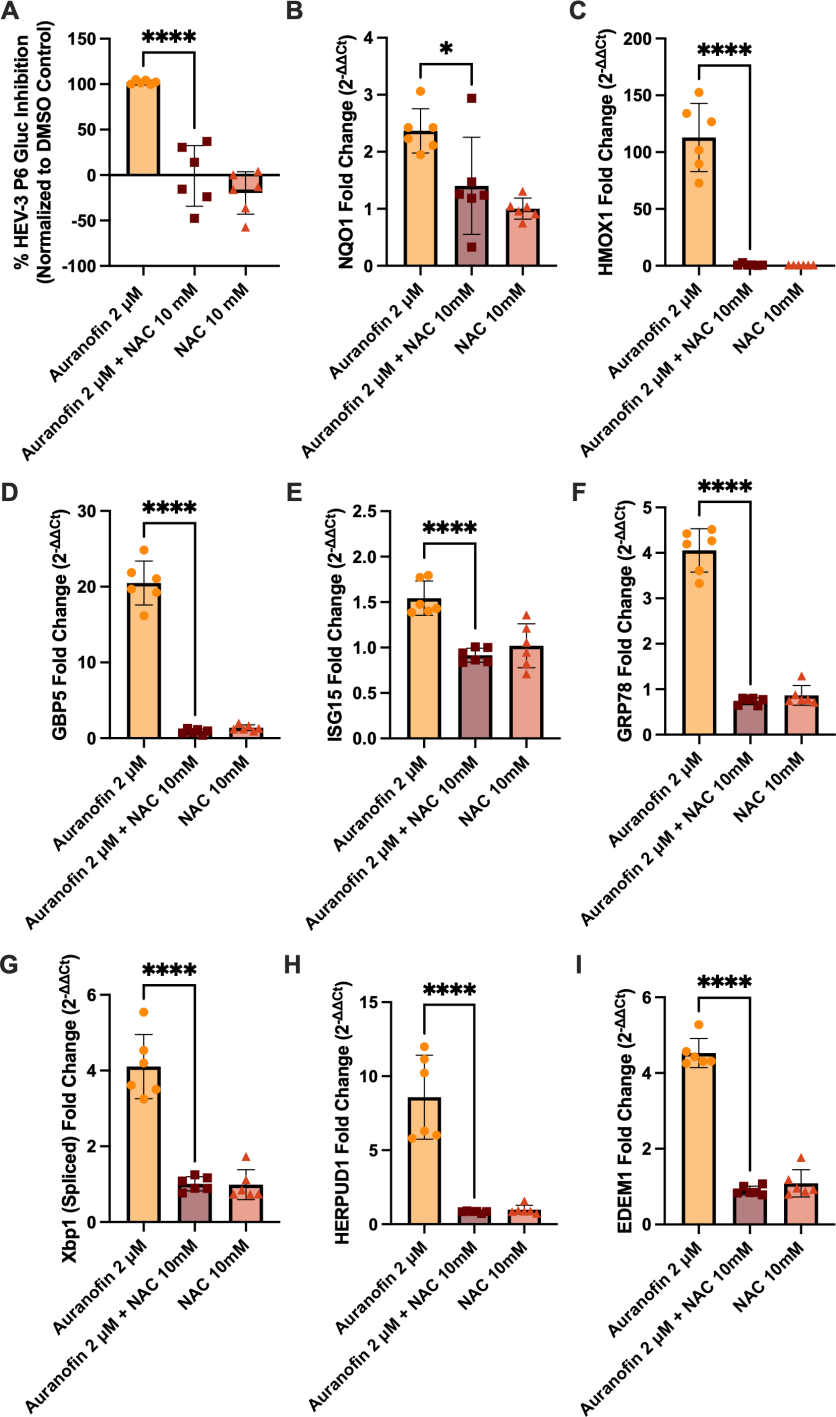
In the presence of HEV-3, auranofin triggers ROS-dependent antioxidant, antiviral, and ER stress responses. (**A**) The effect of 2 μM auranofin in the presence of 10 mM NAC on HEV-3 P6 Gluc production as a proxy for HEV-3 P6 replication 24 h post-compound application. Data is background-subtracted and normalized to the DMSO control. (**B–I**) Transcriptional modulations measured by RT-qPCR following 24-hour treatment with 2 μM auranofin and/or 10 mM NAC during HEV-3 P6 Gluc infection. Data are presented as fold change of ROS- and Nrf2-related genes (NQO1 and HMOX1) (**B and C**), IFN-stimulated genes (GBP5 and ISG15) (**D and E**), ER stress response genes (GRP78 and Xbp1) (**F and G**), and ERAD-associated genes (HERPUD1 and EDEM1) (**H and I**). Data presented as fold change and normalized to the housekeeping gene (GAPDH) and vehicle (DMSO) control. Data were analyzed by one-way ANOVA with Dunnett’s multiple comparisons test (single pooled variance), with data points representing means ± SD. *n* = 6 performed in two independent experiments. * = *P* < 0.05, **** = *P* < 0.0001.

Auranofin treatment can modulate downstream signaling pathways related to oxidative stress, innate immune responses, and inflammation (66–69). To examine the downstream impacts of ROS promotion via auranofin treatment, we performed reverse transcription-quantitative polymerase chain reactions (RT-qPCR) on RNA extracted from HEV-3 P6 Gluc-transfected Huh7-S10-3 cells treated with auranofin and/or NAC for 24 h. At this time point, the antiviral activity of auranofin was robust and was reversed by NAC treatment (Fig. 4A). We first tested the expression of antioxidant defense genes associated with ROS-induced nuclear factor E2-related factor 2 (Nrf2) oxidative stress defense responses (69, 70). NAD(P)H quinone oxidoreductase 1 (NQO1) (Fig. 4B) and heme oxygenase-1 (HMOX1) (Fig. 4C) were significantly upregulated by auranofin treatment, an effect that was reversed by NAC. The upregulation of NQO1 and HMOX1 is consistent with a functional role for ROS activation of the Nrf2 pathway by auranofin. In parallel, guanylate-binding protein 5 (GBP5) (Fig. 4D) and interferon-stimulated gene 15 (ISG15) (Fig. 4E) were also upregulated by auranofin treatment and reversed by NAC treatment. Both ISG15 and GBP5 are established ISGs and antiviral effectors against multiple viruses (71–75), demonstrating that ROS induced by auranofin activates antiviral signaling.

We also examined transcripts associated with endoplasmic reticulum (ER) stress, as ROS promotion is known to disrupt the tightly regulated redox environment of the ER that is required for proper disulfide bond formation and protein folding, leading to the accumulation of misfolded proteins that can induce ER stress (76, 77). We found that ER stress sensors, including glucose-regulated protein 78 (GRP78), also known as BiP (binding immunoglobulin protein) (Fig. 4F), and X-box binding protein 1 (Xbp1) (spliced) (Fig. 4G), were upregulated by auranofin treatment and reversed by NAC. The upregulation of GRP78 and Xbp1 (spliced) is indicative of the activation of the inositol-requiring enzyme 1 alpha (IRE1α) branch of ER stress response, which attempts to resolve ER stress by reducing the ER folding load and decreasing the amount of misfolded proteins (77). We also examined the upregulation of transcripts associated with the ER-associated degradation (ERAD) response toward misfolded proteins. Transcripts of homocysteine-responsive endoplasmic reticulum-resident ubiquitin-like domain member 1 (HERPUD1) or HERP (Fig. 4H) and ER degradation-enhancing alpha-mannosidase-like 1 (EDEM1) (Fig. 4I) were upregulated by auranofin treatment and reversed by NAC. HERPUD1 and EDEM1 are downstream effectors of ER stress that facilitate ERAD and help clear misfolded proteins or target them for degradation (78–80). Taken together, these transcriptional perturbations highlight the downstream functional consequences of auranofin-mediated ROS promotion and provide evidence for potential antiviral mechanisms of action of ROS promotion.

### Auranofin and ribavirin combined treatment exhibits synergistic antiviral effects

**Fig 5:**
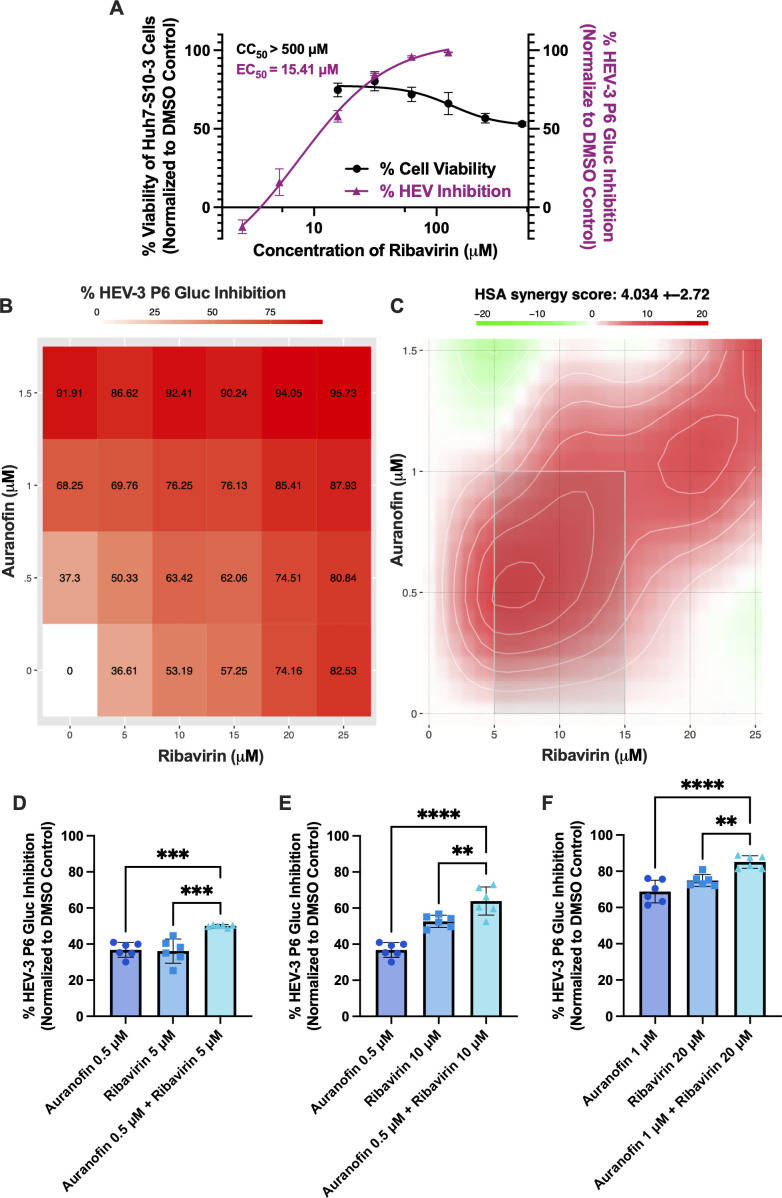
Auranofin and ribavirin combined treatment *in vitro* displays synergistic antiviral activity against HEV-3. (**A**) The effect of ribavirin on cytotoxicity of Huh7-S10-3 cells and antiviral activity against the HEV-3 P6 Gluc replicon. Data collected 72 h post-compound application is background-subtracted and normalized to the vehicle control. X-axis values are log-transformed. The lines represent non-linear regression curves, and data points represent means ± SD; *n* = 3 performed in two independent experiments. (**B and C**) Huh7-S10-3 cells were transfected with HEV-3 P6 Gluc RNA, compounds were applied, and Gluc was quantified from supernatants 72 h post-complication. Resulting data were generated via the SynergyFinder web application (version 3.0). (**B**) Dose-response HEV-3 P6 Gluc inhibition matrix of the combined concentrations of auranofin and ribavirin tested at a range between 0–1.5 μM and 0–25 μM, respectively. The average inhibition of HEV replication is presented for all tested compound combinations and for auranofin and ribavirin treatment alone. (**C**) Synergy map generated via the HSA model. The overall HSA synergy score is depicted as 4.034 ± 2.72. (**D–F**) Graphs depicting the concentration combinations that displayed synergy (synergy scores over 10): (**D**) auranofin 0.5 μM + ribavirin 5 μM, (**E**) auranofin 0.5 μM + ribavirin 10 μM, and (**F**) auranofin 1 μM + ribavirin 20 μM. Data in panels B–F are background-subtracted and normalized to the appropriate vehicle control. A one-way ANOVA with Dunnett’s multiple comparisons test (single pooled variance) was performed for panels D–F. The data points represent means ± SD; *n* = 6, performed in two independent experiments. ** = *P* < 0.01, *** = *P* < 0.001, **** = *P* < 0.0001.

Since the antiviral activity of auranofin and ribavirin is likely through different mechanisms, we hypothesized that a combined treatment would exert synergistic antiviral activity (81). This is an important consideration because multiple ribavirin-resistant mutants have been identified in patients, and a combined antiviral treatment could prevent the development and further expansion of ribavirin-resistant HEV strains (82), representing a more effective treatment option. We first assessed the cytotoxicity and antiviral activity of ribavirin to determine the optimal concentration range for combined treatment testing. Ribavirin displays stable cell viability in Huh7-S10-3 cells, with a CC_50_ > 500 μM, and shows dose-dependent antiviral activity with an EC_50_ of 15.41 μM (Fig. 5A).

To determine the potential synergistic antiviral activity, auranofin and ribavirin were tested in combination against the HEV-3 P6 Gluc replicon at concentrations ranging from 0 to 1.5 μM and 0 to 25 μM, respectively. Antiviral data were uploaded to the SynergyFinder web application to identify synergy, defined as a synergy score greater than 10. The highest single agent (HSA) model was used to determine whether the combined effect of the two drugs exceeds the sum of their individual effects. This provides a stronger weight toward lower concentrations, which are less likely to display toxicity (83). We observed a dose-response to combined auranofin and ribavirin treatments against HEV-3 P6 Gluc for each tested concentration combination (Fig. 5B), with average inhibition scores shown. Based on the HSA model, three of the concentration combinations tested in the study display a synergy score over 10, which can be interpreted as 10% of response beyond the individual compound effects (83) (Table S1), resulting in an overall HSA synergy score of 4.034 ± 2.72 (Fig. 5C). The three combinations that display synergy are 0.5 μM auranofin + 5 μM ribavirin with a synergy score of 13.03 (Fig. 5D), 0.5 μM auranofin + 10 μM ribavirin with a synergy score of 10.24 (Fig. 5E), and 1 μM auranofin + 20 μM ribavirin with a synergy score of 11.26 (Fig. 5F). Overall, this data demonstrates that auranofin and ribavirin display synergistic antiviral activity at select concentrations *in vitro*.

## DISCUSSION

HEV infection is associated with serious clinical conditions, including chronic infection, fulminant hepatic failure, and neurological sequelae, which require effective antiviral intervention. Due to the emergence of HEV strains resistant to ribavirin (37, 38), the current off-label standard of care, it is essential to identify more effective antivirals that act through different mechanisms. Since auranofin is an FDA-approved, ROS-promoting compound shown to be effective against several other viruses (46, 48, 49), we investigated its potential as an antiviral against HEV while seeking to define its mechanism of action. We demonstrated that auranofin exhibits dose-dependent antiviral activity against HEV replicons and fully infectious reporter virus systems of two different HEV genotypes (HEV-1 and HEV-3), as well as an HEV-3 mutant associated with ribavirin treatment failure. We further showed that auranofin and ribavirin displayed synergistic antiviral activity, supporting a combined therapeutic strategy to enhance antiviral efficacy and prevent antiviral resistance. We identified that ROS promotion was necessary for the antiviral activity of auranofin, as reversing ROS through two distinct mechanisms reversed its antiviral effects. We then established ROS as sufficient for driving anti-HEV activity by showing that DAAO, an enzyme that produces H_2_O_2_, had robust anti-HEV activity with no toxicity at the doses tested. These findings were functionally supported by upregulated transcripts associated with ROS promotion, Nrf2 activation, ISG induction, ER stress, and ER-associated degradation of misfolded proteins. Altogether, this work has revealed the promotion of ROS as a novel, host-directed antiviral strategy against HEV and highlights the potential for combinational treatment of auranofin with ribavirin to induce synergistic antiviral effects via differential mechanisms. These findings also redefine redox modulation as a deliberate antiviral strategy, establishing a foundation for ROS-promoted, host-targeted antiviral design.

Auranofin is well-studied and has both anti-cancer and broad-spectrum anti-pathogen effects (45). Auranofin’s anti-cancer activity has been linked to the accumulation of ROS via the inhibition of the redox enzymes, GPx and TrxR, which play important roles in the glutathione and thioredoxin redox systems, respectively (55, 84, 85). These disulfide reductase systems are antioxidant in nature and function to maintain ROS homeostasis within cells (86). Numerous studies present the connection between the inhibition of these enzymes, the upregulation of ROS, and auranofin’s anti-cancer effects (57, 87, 88). This connection is supported by the finding that NAC, a ROS inhibitor, and auranofin co-treatment reverse the toxicity of auranofin (58, 87, 88). Here, we found that NAC also reversed auranofin’s antiviral activity, a novel finding in the investigation of auranofin’s antiviral mechanism of action.

Because auranofin targets host cells to modulate redox pathways, the level of auranofin-dependent ROS production varies across cell lines (89). Levels of ROS promotion are also dependent upon the method of perturbation. For example, auranofin and DAAO induce ROS through different mechanisms, which likely contribute to differences in ROS levels. DAAO breaks down D-amino acids into H_2_O_2_ (65), while auranofin has been shown to lead to the production of H_2_O_2_ (55, 90) and superoxide (O₂•⁻) (58, 87, 91). This highlights a limitation of this study, as the H_2_DCFDA ROS probe used to measure ROS levels is non-specific. While this probe is commonly used and accepted in auranofin literature (58, 87, 92, 93), the future use of more specific ROS probes, such as HyPer7 (94) or Amplex UltraRed (55), will help to flesh out the type of ROS responsible for auranofin’s antiviral activity.

To mitigate this limitation, we employed an orthogonal approach to investigate ROS-induced transcriptional pathways, providing evidence for ROS-promoted antiviral activity against HEV. The finding that HMOX1 and NQO1 are upregulated during auranofin treatment (Fig. 4B and C) and reversed by NAC treatment indicates that ROS-induced signaling pathways related to oxidative stress defense are likely activated. HMOX1 and NQO1 are antioxidant defense genes that are induced following the activation of Nrf2, a transcription factor that binds to antioxidant response elements (AREs), resulting in increased antioxidant activity in response to oxidative stress (95). It is also well established that auranofin activates Nrf2 and ARE pathways (66, 96, 97), and several viruses have been shown to alter Nrf2 activity (98–100). Thus, modulation of Nrf2 signaling pathways has been suggested as a potential antiviral strategy. In parallel with these findings, genes associated with IFN-induced antiviral activity, GBP5, and ISG15 were also upregulated by auranofin treatment. ISG15 is a ubiquitin-like protein that is strongly induced by type I IFN and correlates with antiviral activity against various viruses, including HEV (71, 72, 101, 102). GBP5 is an interferon-induced GTPase that plays a crucial role in defense against various pathogens (73, 103–105). The induction of ISGs provides further support that auranofin-mediated ROS promotion activates antiviral signaling pathways that may contribute to its activity against HEV.

We also examined the modulation of transcriptional pathways associated with ER stress, as ROS promotion is known to induce stress on the ER through the accumulation of misfolded proteins (106). ROS accumulation in the ER can break disulfide bonds, induce oxidative damage, and cause non-native disulfide bonds to form, leading to misfolding of proteins that accumulate and induce ER stress (107). Our results indicate that genes in the IREα branch of the unfolded protein response (UPR), GRP78, and Xbp1 (spliced) were upregulated, suggesting activation through auranofin-mediated ROS promotion. GRP78, also known as BiP, is an ER chaperone that associates with and inhibits ER stress sensor proteins, such as IRE1α, under redox-balanced conditions (108, 109). However, during ER stress and the accumulation of misfolded proteins, GRP78 dissociates from IRE1α, which then splices Xbp1, a transcription factor that regulates genes associated with maintaining ER homeostasis (110, 111). The UPR also consists of the ER-associated degradation (ERAD) of misfolded proteins. The upregulation of HERPUD1 and EDEM1 suggests that ERAD is engaged as HERPUD1 helps to recruit and stabilize the ubiquitin-proteasome degradation system, while EDEM1 recognizes and marks misfolded proteins for ERAD (78, 80, 112, 113). The finding that ER stress, UPR, and ERAD-associated transcripts are reversed by NAC suggests that they are activated by ROS, which may contribute to the antiviral activity of ROS. Altogether, these transcriptional modulations support a model in which auranofin-mediated ROS promotion shapes downstream transcriptional responses, including oxidative stress, IFN signaling, and ER stress-related pathways that are associated with antiviral activity.

Many viruses alter ROS levels and ROS-related signaling pathways, making modulation of ROS a promising antiviral strategy (114, 115). Several studies have previously reported auranofin’s antiviral activity against other viruses, although the suggested mechanisms vary. For example, auranofin was shown to be effective against chikungunya virus *in vivo*. The authors speculated that oxidative folding pathways were involved, but mechanistic studies were not performed to corroborate this (46). Auranofin also impacts human immunodeficiency virus type 1 (HIV-1) reservoirs by inducing a pro-apoptotic effect via a burst of ROS in HIV-infected CD4+ T cells, which are typically unaffected by antiretroviral therapy (ART) (48). During a randomized clinical trial, auranofin in conjunction with ART decreased total integrated HIV-1 DNA compared to ART alone, suggesting its efficacy against HIV-1 infection in humans (47). Auranofin also inhibits SARS-CoV-2 replication and reduces cytokine production, supporting both antiviral and immunomodulatory effects (49). Auranofin and similar analogs have also been linked to anti-protease activity against SARS-CoV-2 (50). Collectively, these studies suggest that auranofin can inhibit diverse viruses, although ROS involvement has not previously been demonstrated during acute infection.

There is also evidence that ROS themselves can exert antiviral effects against viruses. For instance, treatment with H_2_O_2_ inhibits hepatitis C virus replication, an effect reversed by NAC (116). The viral components of phages, including proteins, nucleic acids, and lipids, can also be directly damaged by different types of ROS, resulting in their inactivation (117). Consistent with this, our findings show that DAAO, which preferentially promotes H_2_O_2_, mediates anti-HEV effects, supporting the concept that ROS promotion may be a viable broad-spectrum antiviral strategy.

Further research is needed to investigate the antiviral effects of auranofin and ROS against HEV. While we validated the antiviral effects of auranofin against two important genotypes of HEV (HEV-1 and HEV-3) *in vitro*, future studies should validate auranofin’s efficacy against HEV using an animal model. This represents a challenge because, although immunocompetent small animal models of HEV infection are available, working with them requires overcoming obstacles related to genotype specificity, the lack of recapitulated disease, and high costs associated with animal care and housing (118). Additional work with auranofin could also explore its efficacy against chronic HEV infections, as ribavirin resistance arises in individuals with chronic HEV infections and liver failure (119). Host-targeting antivirals, such as auranofin, are expected to retain efficacy and prevent the emergence of viral resistance (120).

In conclusion, we have identified auranofin’s dose-dependent antiviral activity *in vitro* against two different genotypes of HEV and an HEV mutant associated with ribavirin treatment failure. These antiviral effects were mediated by ROS, as ROS inhibitors reversed the antiviral effects and ROS promotion caused by auranofin treatment. We demonstrated that ROS promotion alone was sufficient for antiviral activity using the ROS promoter DAAO, which displayed strong antiviral activity with no toxicity at the tested doses. This finding further highlights ROS promotion as a novel and promising antiviral strategy against HEV. The ROS-dependent antiviral activity of auranofin was also supported via promotion of transcripts associated with oxidative defense, IFN-stimulated antiviral pathways, and ER stress. Finally, a combined treatment of auranofin and ribavirin demonstrated that synergistic antiviral activity can be achieved at specific concentrations, providing combinational drug therapeutic potential to enhance antiviral activity and minimize ribavirin resistance. Altogether, these data support the conclusion that auranofin inhibits HEV replication by promoting the production of ROS. While various studies have shown auranofin’s antiviral activity, this is the first study to mechanistically link its antiviral activity to its promotion of ROS. Overall, this work lays the foundation for further studies to explore the mechanisms of ROS-promoted antiviral activity, which may represent a broad-spectrum antiviral strategy for treating emerging and re-emerging viral infections. Importantly, HEV-3 has been identified by the WHO as a prototype pathogen, meaning that insights gained from antiviral research against HEV can inform strategies to combat similar and emerging pathogens. Thus, our findings not only advance therapeutic options for treating HEV but also contribute to the broader goal of identifying broad-spectrum antiviral strategies for future outbreaks of novel viruses.

## MATERIALS AND METHODS

### Cell culture

Huh7-S10-3 cells, a sub-clone of human hepatocyte cellular carcinoma cells (Huh7) (121), kindly provided by Suzanne U. Emerson (NIAID, NIH, Bethesda, MD), were cultured in Dulbecco’s modified Eagle’s medium (DMEM) (Corning) with high glucose, L-glutamine, and sodium pyruvate supplemented with 10% fetal bovine serum (FBS), 1% non-essential amino acids, 0.1% gentamicin sulfate, and 25 mM HEPES (herein called DMEM-10). The cells were incubated at 37°C with 5% CO_2_.

### Compounds

Auranofin, kindly provided by Veronica Ghini (Resonance Magnetic Center, Italy), was prepared in dimethyl sulfoxide (DMSO) (Sigma Aldrich) to a stock concentration of 10 mM. Additional auranofin stocks (Selleckchem) were also prepared to 10 mM in DMSO. Ribavirin (Thermo Fisher Scientific) was prepared in molecular grade water to a concentration of 40.95 mM. N-acetylcysteine (NAC) (Thermo Fisher Scientific) was prepared fresh in molecular-grade water to varying concentrations at pH 8, with the addition of sodium hydroxide (NaOH). Dithiothreitol (DTT) (VWR) was purchased at a concentration of 1 M. D-amino acid oxidase (DAAO) (Millipore-Sigma) was prepared in water to a concentration of 22 mg/mL.

### HEV indicator replicons and infectious clones

The HEV-1 Sar55 *Gaussia* luciferase (Gluc) indicator replicon was constructed using the HEV-1 Sar55 strain backbone (GenBank accession no. AF444002) (122), in which a portion of ORF2 was replaced with the *Gaussia* luciferase gene (123). Similarly, the HEV-3 P6 Gluc indicator replicon was developed from the HEV-3 strain Kernow-C1/P6 (designated as P6) backbone (GenBank accession no. JQ679013) that was serially passaged six times in cell culture (124, 125). Additionally, a G1634R mutation was introduced into the HEV-3 P6 Gluc construct, based on prior findings showing that this mutation significantly enhances HEV-3 replication *in vitro* and promotes ribavirin resistance *in vivo* (54). This mutant was constructed using a site-directed mutagenesis system, as previously described (126). An HEV-1 infectious luminescence reporter virus Sar55 (Hib), which has a HiBiT tag in the C-terminal of ORF2 of the wild-type Sar55 strain, was generated as previously described (51). The HEV-3 P6 Gluc indicator replicon was kindly provided by Dr. Sue Emerson of the National Institute of Allergy and Infectious Diseases at NIH. The HEV-1 Sar55 Gluc indicator replicon was generously provided by Dr. Alexander Ploss of Princeton University, Princeton, NJ.

### Construction and assembly of HEV-3 P6 HiBiT-expressing infectious clone

An HEV-3 infectious luminescence reporter virus P6(Hib) was generated using the wild-type (WT) HEV-3 P6 infectious clone backbone via a bacterial-free cloning approach to incorporate a HiBiT tag into the C-terminal of the ORF2 (53). Briefly, a plasmid containing a glycine-serine linker (24 nt), HiBiT (33 nt), two stop codons (6 nt), and the last 60 nucleotides of ORF2 was synthesized by Twist Bioscience, as previously described by Nagashima et al. and Tian et al. (51, 52). This synthesized sequence also contained overlapping regions in ORF2 and the C-terminus of WT HEV-3 P6 genome that are important for assembly. The insert sequence was amplified via repliQa HiFi ToughMix (Quantabio), and the WT HEV-3 P6 genome was amplified in two fragments using Platinum SuperFi II (Invitrogen). The fragments were gel purified using the NucleoSpin Gel and PCR Clean-up Kit (Macherey-Nagel) and quantified by Qubit. Fragments were assembled in equimolar ratios using the NEBuilder HiFi DNA Assembly Master Mix (New England Biolabs), followed by exonuclease and DpnI treatment to remove unassembled components and residual plasmid template.

### Replicon and infectious clone amplification, *in vitro* transcription, and transfection

Replicon, infectious clone, or HiFi assembled plasmids were amplified via rolling circle amplification (RCA) as previously described (127). The RCA product was then linearized with the restriction enzyme MluI (for P6-based) or BglII (for Sar55-based) (New England Biolabs). The linearized product was purified using SPARQ PureMag Beads (Quantabio), and *in vitro* transcription was performed with the mMESSAGE mMACHINE T7 Transcription Kit (Thermofisher Scientific) to produce capped, infectious RNA. Viral RNA was transfected via the jetMESSENGER mRNA transfection reagent (Polyplus) into Huh7-S10-3 cells and incubated with 5% CO_2_ at 37°C.

### Cytotoxicity assay

Huh7-S10-3 cells were plated at 5,000 cells/well in a 96-well plate and incubated at 37°C with 5% CO_2_. Compound dilutions were prepared in DMEM-10. The vehicle (DMSO or H_2_O) was also prepared to corresponding concentrations to serve as a control. Growth media was removed from the cells and replaced with the same volume of each compound dilution. Cells were incubated for 72 h before CellTiter 96 AQueous One Solution Reagent was added to each well as recommended by the manufacturer’s protocol (Promega CellTiter 96 AQueous One Solution Cell Proliferation Assay). Plates were incubated at 37°C with 5% CO_2_ for 2–4 h. After incubation, the absorbance was measured at 490 nm using an Infinite M plate reader. Viability was calculated by subtracting media-only absorbance values and normalizing to the appropriate vehicle control. Cytotoxic concentration 50% (CC_50_) values were generated via the ED50 Plus v1.0 software (128).

### HEV antiviral assays

Huh7-S10-3 cells were plated at 5,000 cells/well in a 96-well plate. Cells were transfected with HEV replicon- or infectious clone-derived RNA as described above. After a 4-hour incubation, compound dilutions were prepared in DMEM-10. The vehicle (DMSO or H_2_O) was also prepared to a corresponding concentration to serve as a control. Growth media was removed from the cells and replaced with the same volume of each compound dilution. For the HEV-3 P6 Gluc replicon and infectious reporter virus P6(Hib) testing, cells were incubated with the compounds for 72 h. For HEV-1 Sar55 Gluc replicon and infectious reporter virus Sar55(Hib) testing, cells were incubated with the compounds for 7 days. *Gaussia* luciferase expression was quantified from the supernatant via the Pierce *Gaussia* luciferase glow assay kit (Thermofisher Scientific). HiBiT activity in the supernatant of HEV-1 Sar55(Hib) and HEV-3 P6(Hib) transfected cells was quantified using the Nano-Glo HiBiT Extracellular Detection System (Promega). Luminescence was measured on an Infinite M Plex multimode microplate reader for both Gluc and HiBiT activities. Antiviral activity was calculated by subtracting the negative transfection-only luminescence values and normalizing to the appropriate vehicle control values. The half-maximal effective concentration (EC_50_) values were generated via ED50 Plus v1.0 software (128) for select experiments.

### ROS quantification

Huh7-S10-3 cells were plated in 24-well plates to 50,000 cells/well. Compounds were prepared in DMEM-10 to the desired concentrations. The 2',7'-dichlorodihydrofluorescein diacetate (H_2_DCFDA) (Fisher Scientific) probe was also added to each compound dilution at a final concentration of 10 μM. Compound dilutions were then applied to the cells, and the plates were incubated for 30 min at 37°C with 5% CO_2_. The cells were then trypsinized, and DMEM-10 was added to create a single-cell suspension. Cells from two wells were pooled for each sample and pelleted at 300 × *g* for 5 min at 4°C. Then, the cells were washed with PBS, re-pelleted, and finally resuspended in 100 μL of PBS. Samples were analyzed by flow cytometry using the FACSAria Fusion Flow cytometer (BD Biosciences), and the median fluorescent peak was recorded. The data were then analyzed by normalization to the appropriate vehicle control: DMSO for auranofin and media alone for DAAO. Since DMSO increases ROS levels (64), normalization was expected to differ between auranofin and DAAO treatments.

### RNA extraction and reverse transcription‐quantitative polymerase chain reaction (RT‐qPCR)

Huh7-S10-3 cells were plated in 24-well plates at 50,000 cells/well. Cells were transfected with HEV-3 P6 Gluc RNA, and compounds (auranofin at 2 μM and NAC at 10 mM) were prepared and applied to the cells 4 h post-transfection. Plates were incubated for 24 h at 37°C with 5% CO_2_. After incubation, 500 μL of TRIzol reagent (ThermoFisher) was added to the wells. RNA was extracted following the manufacturer’s protocol and stored at −80°C until use. RT-qPCR was performed on the extracted RNA using the Luna Universal One‐Step RT‐qPCR Kit with SYBR Green (New England Biolabs). Primers were ordered from Integrated DNA Technologies (IDT) and are listed in Table S2 (129–132). RT-qPCR reactions were performed using a QuantStudio Real-Time PCR System with the following reaction conditions: reverse transcription at 55°C for 20 min, reverse transcription deactivation at 95°C for 5 min, followed by 40 cycles of denaturation at 95°C for 10 s and annealing/extension at 60°C for 60 s. A melt curve was generated at 60°C to 95°C with 0.5°C increments and an interval of 0.05 s. Relative gene expression was calculated by normalizing the Ct values of genes of interest to the housekeeping gene, GAPDH.

### Synergy analysis

Huh7-S10-3 cells were plated at 5,000 cells/well in a 96-well plate. Cells were transfected with HEV-3 P6 Gluc RNA, and compounds were prepared in DMEM-10 to the desired concentrations. Auranofin was tested at concentrations of 0, 0.5, 1, and 1.5 μM, and ribavirin was tested at concentrations of 0, 5, 10, 15, 20, and 25 μM. Each concentration was tested alone and in combination with auranofin and ribavirin, with all possible concentration combinations being tested. Compounds were applied, and 72 h later, Gluc expression was quantified from the supernatant. Antiviral activity was calculated by subtracting the negative transfection only luminescence values and normalizing to the appropriate vehicle control values. Data was uploaded to the SynergyFinder web application (version 3.0) (83). The synergy between auranofin and ribavirin was determined using the HSA model. This model determines if the combined effect of two drugs exceeds the sum of their individual effects; if so, then a synergistic effect exists. Combinations with a synergy score greater than 10 were graphed independently for statistical analysis.

### Statistical analysis

Statistical analyses were performed using Graphpad Prism 10. For initial cytotoxicity and antiviral testing, tested compound concentrations were converted to a log scale, and non-linear regression ([log inhibitor] vs normalized response) was performed. To determine EC_50_ and CC_50_ values, data were generated via ED50 Plus v1.0 software (128). For all other antiviral and ROS testing, data were analyzed via one-way analysis of variance (ANOVA) using Dunnett’s test for multiple comparisons. Combined testing of auranofin and ribavirin was analyzed via the SynergyFinder web application (version 3.0) (83). Select compound combinations were analyzed via one-way ANOVA as previously mentioned.

## Data Availability

All data needed to evaluate the conclusions in the paper are present in the paper and supplemental material.

## SUPPLEMENTAL MATERIAL

Fig. S1 jvi.01917-25-s0001.pdf Auranofin demonstrates dose-dependent antiviral activity against HEV-3 P6 Gluc at non-toxic concentrations.

Table S1 jvi.01917-25-s0002.pdf Synergy scores from combined auranofin and ribavirin treatment.

Table S2 jvi.01917-25-s0003.pdf Sequences of primers used for RT-qPCR.

ASM does not own the copyrights to Supplemental Material that may be linked to, or accessed through, an article. The authors have granted ASM a non-exclusive, world-wide license to publish the Supplemental Material files. Please contact the corresponding author directly for reuse.
