# Supplementary material for: Reactive oxygen species promotion drives auranofin’s antiviral activity against hepatitis E virus
Source: J Virol. 2025 Dec 9;100(1):e01917-25. doi: 10.1128/jvi.01917-25 (PMC12817905; doi:10.1128/jvi.01917-25)
Supplement: Fig. S1 — Auranofin demonstrates dose-dependent antiviral activity against HEV-3 P6 Gluc at non-toxic concentrations. [file jvi.01917-25-s0001.pdf]

A

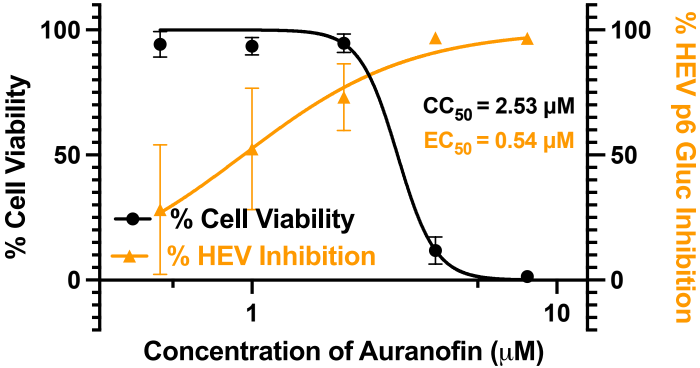

Supplementary Figure 1. Auranofin demonstrates dose-dependent antiviral activity against HEV-3 P6 Gluc at non-toxic concentrations. Cell viability (black) and antiviral activity (orange) were measured 72 hours post auranofin treatment on Huh7-S10-3 cells. Cell viability was measured via MTS assay, and antiviral activity was measured via quantification of secreted Gluc as a proxy for measuring replication of HEV-3 P6 Gluc. Data is background subtracted and normalized to the DMSO vehicle control. X-axis values are log transformed. The lines represent non-linear regression curves, and data points represent means ± SD; n=6 performed in two independent experiments.
