# Supplementary material for: Reactive oxygen species promotion drives auranofin’s antiviral activity against hepatitis E virus
Source: J Virol. 2025 Dec 9;100(1):e01917-25. doi: 10.1128/jvi.01917-25 (PMC12817905; doi:10.1128/jvi.01917-25)
Supplement: Table S1 — Synergy scores from combined auranofin and ribavirin treatment. [file jvi.01917-25-s0002.pdf]

Supplementary Table 1. Synergy Scores for Combined Auranofin and Ribavirin Treatments

Drug1	Drug2	Conc.1	Conc.2	Relative inhibition	Synergy	Conc. Unit	SD	SEM
Auranofin	Ribavirin	0	0	0	0	μM	0	0
Auranofin	Ribavirin	0.5	0	37.295	0	μM	4.08034	1.665792
Auranofin	Ribavirin	1	0	68.25	0	μM	6.282231	2.56471
Auranofin	Ribavirin	1.5	0	91.91	0	μM	4.445108	1.814708
Auranofin	Ribavirin	0	5	36.615	0	μM	6.694925	2.733192
Auranofin	Ribavirin	0.5	5	50.325	13.03	μM	0.739613	0.301946
Auranofin	Ribavirin	1	5	69.76	1.51	μM	7.767702	3.171151
Auranofin	Ribavirin	1.5	5	86.62	-5.29	μM	5.872003	2.397235
Auranofin	Ribavirin	0	10	53.19	0	μM	3.369196	1.375468
Auranofin	Ribavirin	0.5	10	63.425	10.235	μM	7.79889	3.183883
Auranofin	Ribavirin	1	10	76.245	7.995	μM	5.522929	2.254726
Auranofin	Ribavirin	1.5	10	92.415	0.505	μM	5.817225	2.374872
Auranofin	Ribavirin	0	15	57.25	0	μM	5.806591	2.370531
Auranofin	Ribavirin	0.5	15	62.065	4.815	μM	3.711214	1.515097
Auranofin	Ribavirin	1	15	76.13	7.88	μM	6.339548	2.58811
Auranofin	Ribavirin	1.5	15	90.24	-1.67	μM	5.473436	2.234521
Auranofin	Ribavirin	0	20	74.16	0	μM	3.264069	1.332551
Auranofin	Ribavirin	0.5	20	74.51	0.35	μM	2.381929	0.972419
Auranofin	Ribavirin	1	20	85.415	11.255	μM	3.433568	1.401748
Auranofin	Ribavirin	1.5	20	94.045	2.135	μM	5.470919	2.233494
Auranofin	Ribavirin	0	25	82.525	0	μM	1.852627	0.756332
Auranofin	Ribavirin	0.5	25	80.84	-1.685	μM	2.109585	0.861235
Auranofin	Ribavirin	1	25	87.93	5.405	μM	4.61682	1.884809
Auranofin	Ribavirin	1.5	25	95.73	3.82	μM	4.451174	1.817184

*Yellow highlighting denotes combinations with synergy scores greater than 10.
