# Supplementary material for: Reactive oxygen species promotion drives auranofin’s antiviral activity against hepatitis E virus
Source: J Virol. 2025 Dec 9;100(1):e01917-25. doi: 10.1128/jvi.01917-25 (PMC12817905; doi:10.1128/jvi.01917-25)
Supplement: Table S2 — Sequences of primers used for RT-qPCR. [file jvi.01917-25-s0003.pdf]

Supplementary Table 1. Primers for RT-qPCR

RT-qPCR Primers	Sequence (5'-3')	Reference
Human NQO1 – For.	CCTGCCATTCTGAAAGGCTGGT	Origene (NM_000903)
Human NQO1 – Rev.	GTGGTGATGGAAAGCACTGCCT	Origene (NM_000903)
Human HMOX1 – For.	AAGACTGCGTTCCTGCTCAAC	Shi & Su, 2024
Human HMOX1 – Rev.	AAAGCCCTACAGCAACTGTCG	Shi & Su, 2024
Human ISG15 – For.	CTCTGAGCATCCTGGTGAGGAA	Zhang et al., 2017
Human ISG15 – Rev.	AAGGTCAGCCAGAACAGGTCGT	Zhang et al., 2017
Human GBP5 – For.	AGGCCAAAGCAAGGTAGTGA	Qin et al., 2017
Human GBP5 – Rev.	ATGATGCCACCTGGAAGAGT	Qin et al., 2017
Human GRP78 – For.	CTGTCCAGGCTGGTGTGCTCT	Origene (NM_005347)
Human GRP78 – Rev.	CTTGGTAGGCACCACTGTGTTC	Origene (NM_005347)
Human Xbp1 (spliced) – For.	TGCTGAGTCCGCAGCAGGTG	Schadewijk et al., 2011
Human Xbp1 (spliced) – Rev.	GCTGGCAGGCTCTGGGGAAG	Schadewijk et al., 2011
Human HERPUD1 – For.	CCAATGTCTCAGGGACTTGCTTC	Origene (NM_014685)
Human HERPUD1 – Rev.	CGATTAGAACCAGCAGGCTCCT	Origene (NM_014685)
Human EDEM1 – For.	ACGAGCAGTGAAAGCCCTTTGG	Origene (NM_014674)
Human EDEM1 – Rev.	CCACTCTGCTTTCCAACCCAGT	Origene (NM_014674)
